# Identification of Salt-Stress-Induced Genes from the RNA-Seq Data of *Reaumuria trigyna* Using Differential-Display Reverse Transcription PCR

**DOI:** 10.1155/2014/381501

**Published:** 2014-11-26

**Authors:** Zhen-hua Dang, Qi Qi, Hui-rong Zhang, Hao-yu Li, Shu-Biao Wu, Ying-chun Wang

**Affiliations:** ^1^Key Laboratory of Herbage & Endemic Crop Biotechnology and College of Life Sciences, Inner Mongolia University, Hohhot 010021, China; ^2^School of Environmental and Rural Science, University of New England, Armidale, NSW 2351, Australia

## Abstract

Next generation sequencing (NGS) technologies have been used to generate huge amounts of sequencing data from many organisms. However, the correct choice of candidate genes and prevention of false-positive results computed from digital gene expression (DGE) of RNA-seq data are vital when using these genetic resources. We indirectly identified 18 salt-stress-induced *Reaumuria trigyna* transcripts from the transcriptome sequencing data using differential-display reverse transcription PCR (DDRT-PCR) combined with local BLAST searches. Highly consistent with the DGE results, the quantitative real-time PCR expression patterns of these transcripts showed strong upregulation by salt stress, suggesting that these genes may play important roles in *R. trigyna*'s survival under high-salt environments. The method presented here successfully identified responsive genes from the massive amount of RNA-seq data. Thus, we suggest that DDRT-PCR could be employed to mine NGS data in a wide range of applications in transcriptomic studies. In addition, the genes identified in the present study are promising candidates for further elucidation of the salt tolerance mechanisms in *R. trigyna*.

## 1. Introduction

Next generation sequencing (NGS) technology has revolutionized genomic and genetic research. However, several recent reports have demonstrated that while gene expressions obtained by digital gene expression (DGE) analysis were, for the most part, consistent with the results of the validation experiments, some discrepancies also occurred [1–3]. Such discrepancies are often difficult to identify and, to some extent, this has affected the use of high-throughput RNA sequencing (RNA-seq) data to identify candidate genes for further study.

*Reaumuria trigyna* (genus* Reaumuria* Linn, family Tamaricaceae) is an endangered small shrubby and dicotyledonous recretohalophyte [4]. This species is endemic to the eastern Alxa-Western Ordos area, a salinized desert (up to 0.7% salt) in Inner Mongolia, China [5].* R. trigyna* has developed remarkable tolerance to the salinized environment [6, 7], making it a good model to study the mechanisms underlying salinity tolerance. Recently, using Illumina Hiseq 2000 sequencing platform, we sequenced and compared the transcriptomes of control and NaCl-treated* R. trigyna* [8]. Functional annotation and DGE analysis showed that 5032 transcripts were differentially expressed between the two transcriptomes and that 33 metabolic pathways changed significantly in response to salt stress. A method that can rapidly identify salt tolerance genes from the massive amount of RNA-seq data is crucial, because DGE results may be misleading in some circumstances, as previously reported [1–3].

Here, we identified fragments of 18 genes that are responsive to salt stress in* R. trigyna* using differential-display reverse transcription PCR (DDRT-PCR) followed by local BLAST searches against the RNA-seq data to identify the same or longer nucleotide sequences. Finally, the differential expression of these transcripts in response to salt stress was confirmed by quantitative real-time PCR (qPCR). DDRT-PCR proved to be a fast approach to screen candidate genes from massive amounts of RNA-seq data and is a step forward towards better use of the available genetic resources generated by NGS technologies.

## 2. Materials and Methods

### 2.1. Plant Cultivation and Stress Treatment

Plump seeds of* R. trigyna* were selected, immersed in 10% sodium hypochlorite for 15 min, and rinsed three times with sterilized double-distilled water. The seeds were germinated in a 150 mL conical flask containing 40 mL MS medium in the dark for 72 h. The germinated seeds were grown in the same medium at 25°C, 70% relative humidity, and a light/dark cycle of 16 h/8 h, for 15 days. When the seedlings were approximately 10 cm high, they were transferred to a tube containing 50 mL of half-strength Hoagland medium and cultured for another 4 weeks, with a change of medium every 2 days. For DDRT-PCR, leaves were collected from the seedling (control sample) for RNA extraction before NaCl treatment. The roots of the seedlings were then immersed in (1/2)-strength Hoagland's medium containing 400 mM NaCl for 48 h. The leaves were then harvested (stressed sample). For qPCR analysis, leaves were collected from the control seedlings grown under normal conditions. For NaCl treatment, the NaCl concentration was increased stepwise from 100 mM to 400 mM at a rate of 100 mM every 8 h. At each concentration, plants were selected and cultured for a further 48 h before the leaves were harvested. The collected samples were immediately snap-frozen in liquid nitrogen and stored at −80°C for subsequent analyses.

### 2.2. RNA Preparation

Total RNA was extracted using the Plant Plus RNA Reagent (Tiangen, Beijing, China), according to the manufacturer's instructions. The extracted RNA was treated with RNase-free DNase I (Takara Bio Inc., Shiga, Japan) for 45 min at 37°C to remove residual DNA. The RNA quality was evaluated using the ratios of absorbance at 260 and 280 nm in sterile water treated with DEPC, and the RNA integrity was tested by electrophoresis on 1% agarose gels.

### 2.3. Reverse Transcription and DDRT-PCR

First-strand cDNA was synthesized using a Quantiscript RT-PCR kit according to the manufacturer's recommendations (Tiangen, Beijing, China). Each reverse transcription reaction was conducted in a total volume of 20 *μ*L, with 1 *μ*g total RNA, 10 × RT buffer, 2.5 mM each dNTP, 10 *μ*M anchor primer (T11A, T11G, or T11C), and Quant reverse transcriptase. RT reactions were performed at 37°C for 60 min. Amplification of the reverse transcribed RNA (cDNA) was performed in a total volume of 20 *μ*L containing 250 mM dNTPs, 10 *μ*M of the corresponding anchored primer, 10 *μ*M arbitrary primer (Table 1), 2 *μ*L cDNA, and 1 U Transtart Taq DNA polymerase (Transgene, Beijing, China). PCR amplifications were performed in a thermocycler for 35 cycles. Each cycle comprised an initial denaturation for 4 min at 94°C, followed by 35 cycles of 30 s at 94°C, 30 s at 42°C, 1 min at 72°C, and a final extension for 10 min at 72°C. Six percent denaturing polyacrylamide DNA electrophoresis and silver staining were used to identify the differentially displayed transcripts.

### 2.4. Reamplification, Cloning, and Sequencing

A rectangular gel slice containing a target band was excised and soaked in 10 *μ*L sterilized double-distilled water at room temperature for 10 min and then in a water bath at 80°C for 15 min. After a short centrifugation, the liquid was transferred to a clean tube. The extracted DNA was used directly as the template for PCR [9]. The PCR conditions were identical to those used for DDRT-PCR. The reamplified products were visualized on 1% agarose gels and recovered using a DNA gel extraction kit (Tiangen, Beijing, China). All screened fragments were subcloned into vector pMD19-T (Takara Bio Inc., Otsu, Shiga, Japan), and recombinant plasmids were used to transform competent DH5*α* cells (Transgene, Beijing, China). Blue/white screening and PCR were performed to differentiate recombinant clones. Invitrogen (Beijing, China) sequenced the inserted DNA fragments of the positive clones.

### 2.5. Local BLAST

A local BLAST was performed using the BioEdit software (http://www.mbio.ncsu.edu/bioedit/bioedit.html) [10]. To create a local nucleotide database, all the previously assembled* R. trigyna* transcriptome unigenes (65,340) [8] were first formatted using the “create a local nucleotide database file” tool in BioEdit. The database was then searched with the sequences obtained by DDRT-PCR as the query sequences using BLASTN with a cutoff *E*-value ≤1.0*E* − 50.

### 2.6. Confirmation of the Expression Patterns of the Identified Transcripts

Reverse transcription reactions were performed using SuperScript III Reverse Transcriptase (Invitrogen, Grand Island, NY, USA) with approximately 5 *μ*g of total RNA, following the manufacturer's instructions. Primers for qPCR were designed using Primer Premier 5 software (listed in additional file 2 in Supplementary Material available online at http://dx.doi.org/10.1155/2014/381501). *β-Actin* was used as the internal control gene. qPCR was performed on a Qiagen Rotor-Gene Q real-time PCR platform (Qiagen, Hilden, Germany) using SYBR-Green real-time PCR mix (Transgene) to detect the gene expression level. The amplification was performed as follows: initial denaturation at 95°C for 30 s, followed by 45 cycles of denaturation at 95°C for 5 s, and annealing and extension at 55°C for 30 s. The relative expression levels of the genes were normalized to*β-actin* and calculated using the 2^−ΔΔCt^ method. All reactions were performed with three replicates. Statistical analysis was performed using the Pearson correlation test in the SPSS19.0 software. A *P* value < 0.05 was considered statistically significant.

## 3. Results

### 3.1. Identification of Salt-Stress-Induced Transcripts by DDRT-PCR

**Figure 1:**
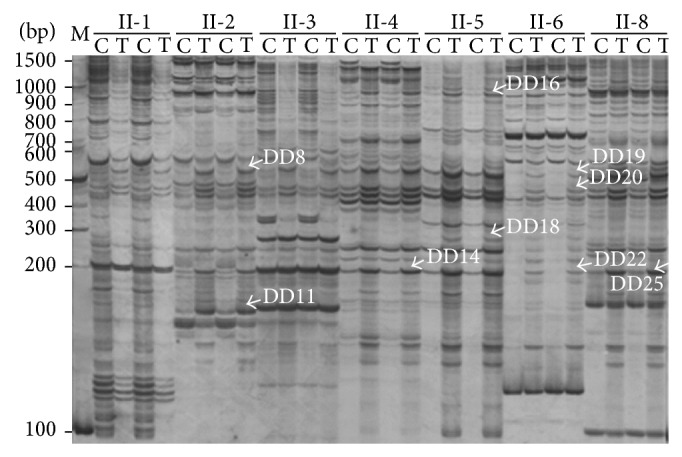
Representative 6% denaturing polyacrylamide gel from a differential-display experiment. Differences between control and NaCl-treated tissues detected by DDRT-PCR with arbitrary primers (1, 2, 3, 4, 5, 6, and 8 in Table 1) in combination with the anchor primer II (Table 1) are shown. II-1, II-2, II-3, II-4, II-5, II-6, and II-8 represent the primer combinations. Lanes C and T contain the PCR products from the control and treated samples, respectively. Lane M contains a 100-bp DNA ladder marker. Arrows indicate the recovered differentially expressed cDNA fragments. Arbitrary primer 7 (Table 1) failed to generate any fragment length polymorphisms.

To identify the salt-stress-induced transcripts and to avoid the isolation of false-positive cDNAs, only bands that clearly showed an increase in band intensity as a result of salt stress were excised from the 6% denaturing polyacrylamide gels. A representative gel from a differential-display experiment is shown in Figure 1. Thirty-three bands were identified and excised from the gels, and 18 were successfully reamplified, subcloned, and sequenced. All the sequenced fragments were of the expected length compared with their positions in the gel and ranged from 155 to 952 bp (Table 2). Nine of the fragments, DD5, DD11, DD14, DD18, DD22, DD25, DD27, DD32, and DD33, were short, with lengths close to or shorter than 300 bp.

### 3.2. Local BLAST Analysis

The 18 fragments were used as queries in a local BLAST search and all of them matched sequences in the local transcriptome database of* R. trigyna* (Table 2). The lengths of DD2, DD3, DD4, DD8, and DD14 were the same as the matched sequences in the RNA-seq data; however, the other 13 fragments were shorter.

The local BLAST searches also revealed the expression patterns of the identified genes. All 18 transcripts were upregulated (fold change ≥ 2) in the salt-stressed samples. DD2, DD14, DD25, and DD30 were highly abundant in both the control and salt-stressed transcriptomes, with reads per kilobase of exon model per million mapped reads (RPKM) values of more than 100. DD3, DD7, DD16, DD22, DD27, and DD32 were identified as strongly salt-induced genes, each with a greater than 10-fold upregulation in the salt-stressed transcriptome compared with that in the control transcriptome. DD4, DD7, DD8, and DD33 were moderately upregulated in the salt-stressed transcriptome compared with that in the control transcriptome.

Of the 18 transcripts, 17 were annotated with a gene description, conserved domains, GO terms, and KEGG metabolic pathways (Table 2; details of the annotations are shown in additional file 1). Clones DD2, DD7, DD11, DD27, and DD32 correspond to mRNAs that encode metal ion binding proteins: farnesylated protein 6, harbinger transposase-derived nuclease, calcium-binding protein, major prion protein, and osmotic stress-induced zinc-finger protein, respectively. Clones DD3, DD22, and DD30 correspond to mRNAs that encode proteins involved in stress-related responses: late embryogenesis abundant (LEA) protein, abscisic stress ripening protein, and cold-regulated protein, respectively. Clones DD16, DD18, and DD25 encode proteins related to secondary metabolites synthesis and metabolism: 12-oxophytodienoate reductase 3 (OPR3), secologanin synthase, and quercetin 3-O-methyltransferase 1 (OMT1). The remaining transcripts represent mRNAs encoding different categories of proteins: clone DD4 encodes the developmental protein phytocalpain, clone DD5 encodes ATP synthase subunit a, clone DD14 encodes SRC2, clone DD19 encodes sulfate transporter, clone DD20 encodes a serine/threonine-protein kinase, and clone DD33 encodes an exocytosis protein of ATEXO70H7. The DD8 sequence did not match any sequence in the public databases, suggesting that it may represent a gene that is unique to* R. trigyna*. The sequences identified from the RNA-seq data have been deposited in GenBank with the accession numbers KC701475 to KC701492.

### 3.3. Detection of the Expression Patterns by qPCR

**Figure 2:**
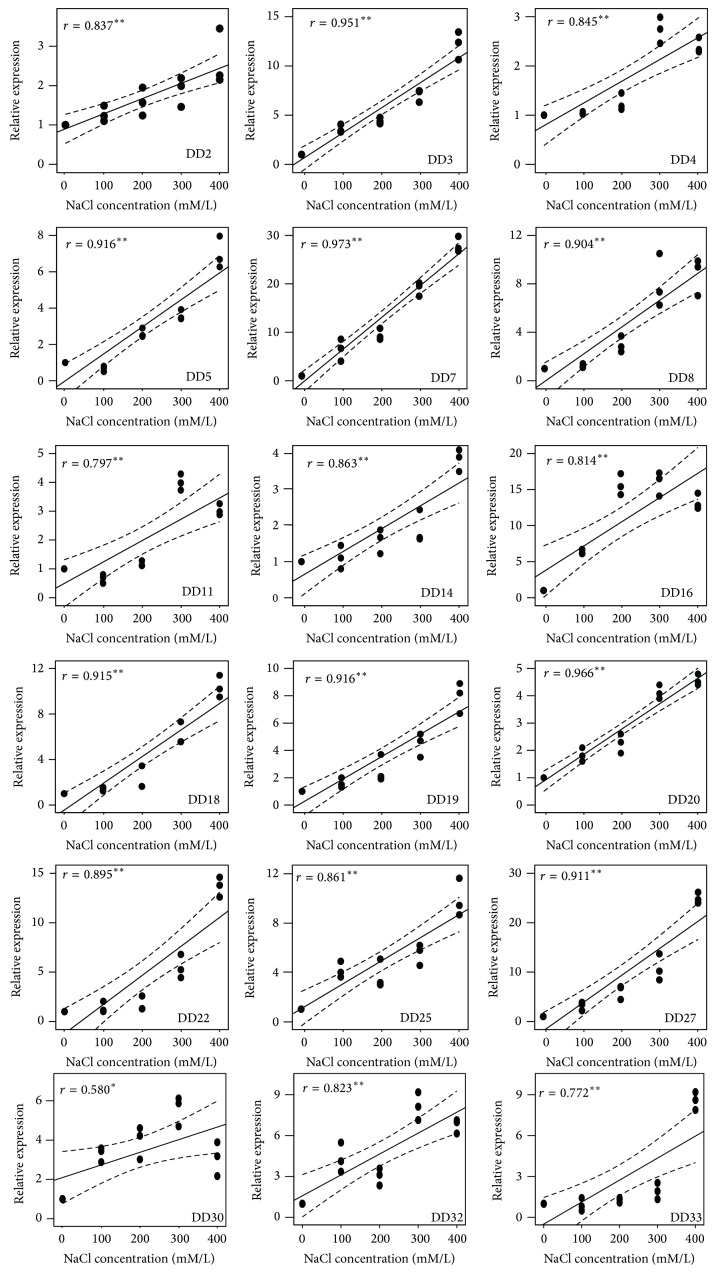
Expression patterns of 18 differentially expressed transcripts detected by qPCR between the control and NaCl-treated samples. The NaCl concentration is shown on the *x*-axis and the relative expression (fold change) is shown on the *y*-axis. Relative expressions of each transcript at one NaCl concentration represent the three independent experimental replicates. Fit lines show the correlation between the gene expression patterns of the differentially displayed transcripts and the NaCl concentrations. Dashed lines indicate 95% confidence intervals. Pearson correlation coefficients (*r*) are shown. ∗∗ and ∗ indicate that the correlations are significant at the 0.01 and 0.05 levels, respectively.

To confirm that the 18 identified gene fragments were indeed salt-stress-induced transcripts, we performed qPCR to measure their expression under different NaCl concentrations. The expressions of these transcripts all increased in response to salt stress. The transcripts of DD2, DD3, DD7, DD8, DD14, DD18, DD19, DD20, DD22, DD25, DD27, and DD33 were upregulated with increasing NaCl concentrations. The expressions of the DD5 and DD11 transcripts were slightly suppressed initially under 100 mM NaCl treatment but subsequently increased with the increasing NaCl concentrations. However, the expression level of DD11 was moderately reduced under 400 mM NaCl. The expression levels of DD16 and DD30 were upregulated immediately upon exposure to NaCl; the highest induction was at 300 mM NaCl, and this reduced slightly when the NaCl concentration was increased to 400 mM. The expressions of these genes were significantly correlated with the NaCl concentration (*P* < 0.05) (Figure 2).

## 4. Discussion

NGS technologies have been used to generate huge amounts of transcriptomic data in many organisms in a very cost-effective and rapid manner. The generated data have then been used to accelerate our understanding of the integrative gene expression profiles between cultivars, tissues, development stages, and stress conditions [11, 12]. However, the methods used to generate and analyze the data are not perfect, and sequencing bias is observed frequently in transcriptome sequencing projects. Therefore, it is important to identify true positive RNA-seq results from the massive amounts of sequencing data.

For the nonmodel species* R. trigyna*, we used the previously sequenced transcriptome data and the available data analysis approaches to investigate gene expression patterns in response to salt stress. In the present study, a conventional DDRT-PCR technique was used to identify salt-stress-induced transcripts that were then used to probe the sequences in the transcriptome database, thereby indirectly identifying true positive genes from the massive amount of RNA-seq data. This approach identified 18 salt-stress-induced transcripts from the transcriptome database of* R. trigyna*. Among the identified transcripts, DD3 was predicted to encode an LEA protein and was significantly upregulated under high salt concentration. LEAs are extremely hydrophilic proteins that are induced by abiotic stresses, such as high salinity, osmotic stress, and freezing [13]. In salt-treated* Oryza sativa*,* Glycine max*, and* Thellungiella salsuginea*, LEA transcripts accumulated in the roots, hypocotyls, and aerial parts of the plants [14–16]. The similarities between the LEA expression profiles in other species and DD3 expression in* R. trigyna* suggest that DD3 may be an important osmolyte that regulates the response of* R. trigyna* cells to the stress posed by saline conditions. DD16 encodes a protein homologous to 12-oxophytodienoate reductase 3 (OPR3), an important enzyme in the alpha-linolenic acid metabolism pathway, which converts 12-oxo-phytodienoic acid to 3-oxo-2-(29-pentenyl)-cyclopentane-1-octanoic acid and then to jasmonic acid. The accumulated jasmonic acid acts as a signaling molecule in multiple stress responses [17]. Therefore, we propose that DD16, which is strongly induced in salt-stressed* R. trigyna*, may play an important role in salt-stress tolerance in this species. DD25 shows sequence similarity to a gene encoding an* O*-methyltransferase (OMT). Plant* O*-methyltransferases are a large family of enzymes that methylate oxygen atoms in a variety of secondary metabolites, mostly phenylpropanoids, flavonoids, and some alkaloids. The methylated products play major roles in stress tolerance, disease resistance, and lignin biosynthesis in plants [18]. Our previously published transcriptomic analysis showed that large numbers of genes were enriched in secondary metabolite biosynthesis pathways, especially the phenylpropanoid and flavonoid pathways, and these pathways were significantly activated in the salt-stressed transcriptome of* R. trigyna* [8]. The results suggested that DD25 could be involved in the accumulation and metabolism of secondary metabolites in* R. trigyna*. In addition, the expression patterns of the identified genes were highly consistent with the DGE results for the transcriptome database as well as with the differential display of electrophoretic bands, which confirmed the applicability of the DDRT-PCR technique for identifying genes of interest from RNA-seq data.

Our combined experimental approach took advantage of the simplicity and sensitivity of the DDRT-PCR technique and overcame the shortcomings related to the high false-positive rate of the RNA-seq approach and the short sequence fragments of DDRT-PCR, which can limit the reliability of the methods [19, 20]. In the present study, only those genes with clear band patterns and obviously upregulated expression levels were identified from the DDRT-PCR results; thus, the problem of high rates of false positives was avoided. Furthermore, the qPCR assay confirmed that the 18 identified transcripts all yielded positive results. Thirteen of these transcripts matched assembled sequences in the local transcriptome database, allowing the sequences of these transcripts to be significantly extended from the short sequences produced from DDRT-PCR amplifications. For example, the DD33 transcript obtained by DDRT-PCR was only 223 bp, but the corresponding gene in the transcriptome assembly was 1246 bp.

## 5. Conclusions

The methods used in the present study represent a novel approach for the mining of RNA-seq data. Despite the limited number of candidate genes identified, this approach significantly reduced the time and effort required to identify the responsive genes in the transcriptome database. Furthermore, our findings provide a valuable resource for in-depth studies of the molecular mechanism underlying salt tolerance in* R. trigyna*.

## Supplementary Material

Additional file 1 provides the detailed annotation information of the 18 identified transcripts.
Additional file 2 provides the primer sequences used for qPCR experiments.

## Figures and Tables

**Table 1:** Primers used in the DDRT-PCR analysis.

Anchor primer (5′-3′)	Arbitrary primer (5′-3′)
(I) AAGCTTTTTTTTTTTG	(1) AAGCTTGATTGCC
(II) AAGCTTTTTTTTTTTA	(2) AAGCTTCGACTGT
(III) AAGCTTTTTTTTTTTC	(3) AAGCTTTGGTCAG
	(4) AAGCTTCTCAACG
	(5) AAGCTTAGTAGGC
	(6) AAGCTTGCACCAT
	(7) AAGCTTAACGAGG
	(8) AAGCTTTTACCGC

Source: GenHunter Corporation, TN, USA.

**Table 2:** Local BLAST analysis based on a database of previously assembled *R. trigyna* transcriptome unigenes.

DD-ID	Length (bp)	Ug-ID	Length (bp)	C-RPKM	T-RPKM	Fold change	Functional description
DD2	757	Ug812	774	136.7	268.7	+2.0	ATFP6
DD3	705	Ug49359	678	49.2	803.9	+16.3	LEA
DD4	469	Ug6492	656	3.1	8.6	+2.8	Phytocalpain
DD5	155	Ug14666	900	13.6	41.3	+3.0	ATP synthase subunit a
DD7	580	Ug29649	909	5.8	194.7	+33.4	Putative nuclease HARBI1
DD8	517	Ug65098	686	9.6	78.4	+8.1	No BLAST hit
DD11	168	Ug5022	1095	43.9	118.8	+2.7	Calcium-binding protein CML42
DD14	212	Ug47465	289	452.2	1697.8	+3.8	SRC2
DD16	952	Ug3621	1287	21.7	499.9	+23.0	OPR3
DD18	273	Ug56616	1037	0.6	12.0	+20	Cytochrome P450
DD19	503	Ug13783	1889	33.1	142.1	+4.3	Sulfate transporter
DD20	469	Ug814	1208	7.8	31.1	+4.0	Protein kinase-like protein
DD22	208	Ug15235	641	37.3	504.6	+13.5	Abscisic stress ripening protein
DD25	208	Ug49313	614	103.6	650.1	+6.3	Quercetin 3-O-methyltransferase 1
DD27	284	Ug17593	660	31.6	463.6	+14.7	PRP27
DD30	451	Ug1371	905	105.5	428.8	+4.1	Cold-regulated protein
DD32	311	Ug37946	1041	19.6	269.3	+13.8	Osmotic stress-induced zinc-finger protein
DD33	223	Ug1734	1246	2.2	31.0	+14.2	EXO70H7

DD: differential displayed fragment obtained by DDRT-PCR; Ug: previously assembled *R. trigyna* transcriptome unigene [8]; RPKM: reads per kilobase of exon model per million mapped reads in the whole transcriptome of *R. trigyna*; C-RPKM: transcript abundance in the control transcriptome; T-RPKM: transcript abundance in the salt-stressed transcriptome; “+”: salt-induced gene. Functional descriptions were obtained by aligning the 18 unigenes against the plant protein dataset of nr, Swiss-Prot/Uniprot protein database, Clusters of Orthologous Groups databases, Gene Ontology database, and Kyoto Encyclopedia of Genes and Genomes database, respectively [8].

## References

[1] Gahlan P., Singh H. R., Shankar R. (2012). *De novo* sequencing and characterization of *Picrorhiza kurrooa* transcriptome at two temperatures showed major transcriptome adjustments. *BMC Genomics*.

[2] Zhou Y., Gao F., Liu R., Feng J., Li H. (2012). *De novo* sequencing and analysis of root transcriptome using 454 pyrosequencing to discover putative genes associated with drought tolerance in *Ammopiptanthus mongolicus*. *BMC Genomics*.

[3] Liu G., Li W., Zheng P. (2012). Transcriptomic analysis of “Suli” pear (*Pyrus pyrifolia* white pear group) buds during the dormancy by RNA-Seq. *BMC Genomics*.

[4] Ma Y. (1989). *Flora Intramongolica (Tomus 3)*.

[5] Zhao Y. (2006). *Vascular Plants in Ordos Plateau*.

[6] Xue Y., Wang Y. (2008). Study on characters of ions secretion from *Reaumuria trigyna*. *Journal of Desert Research*.

[7] Xue Y., Wang Y., Wang T. (2012). Physiological and biochemical mechanisms of an endemic halophyte *Reaumuria trigyna* Maxim. under salt stress. *Acta Botanica Boreali-Occidentalia Sinica*.

[8] Dang Z.-H., Zheng L.-L., Wang J. (2013). Transcriptomic profiling of the salt-stress response in the wild recretohalophyte *Reaumuria trigyna*. *BMC Genomics*.

[9] Wang X., Chang L., Sun Z., Ma H. (2010). Characterization of genes expressed in response to cadmium exposure in the earthworm *Eisenia fetida* using DDRT-PCR. *Ecotoxicology and Environmental Safety*.

[10] Altschul S. F., Madden T. L., Schäffer A. A. (1997). Gapped BLAST and PSI-BLAST: a new generation of protein database search programs. *Nucleic Acids Research*.

[11] Bräutigam A., Gowik U. (2010). What can next generation sequencing do for you? Next generation sequencing as a valuable tool in plant research. *Plant Biology*.

[12] Ekblom R., Galindo J. (2011). Applications of next generation sequencing in molecular ecology of non-model organisms. *Heredity*.

[13] Tunnacliffe A., Wise M. J. (2007). The continuing conundrum of the LEA proteins. *Naturwissenschaften*.

[14] Li X. J., Yang M. F., Chen H., Qu L. Q., Chen F., Shen S. H. (2010). Abscisic acid pretreatment enhances salt tolerance of rice seedlings: proteomic evidence. *Biochimica et Biophysica Acta—Proteins and Proteomics*.

[15] Aghaei K., Ehsanpour A. A., Shah A. H., Komatsu S. (2009). Proteome analysis of soybean hypocotyl and root under salt stress. *Amino Acids*.

[16] Zhang H., Han B., Wang T. (2012). Mechanisms of plant salt response: insights from proteomics. *Journal of Proteome Research*.

[17] Dombrowski J. E. (2003). Salt stress activation of wound-related genes in tomato plants. *Plant Physiology*.

[18] Bureau T., Lam K. C., Ibrahim R. K., Behdad B., Dayanandan S. (2007). Structure, function, and evolution of plant O-methyltransferases. *Genome*.

[19] Liang P., Pardee A. B. (1992). Differential display of eukaryotic messenger RNA by means of the polymerase chain reaction. *Science*.

[20] Sung Y.-J., Denman R. B. (1997). Use of two reverse transcriptases eliminates false-positive results in differential display. *BioTechniques*.

